# Spectrally filtered passive Si photodiode array for on-chip fluorescence imaging of intracellular calcium dynamics

**DOI:** 10.1038/s41598-019-45563-8

**Published:** 2019-06-24

**Authors:** Zheshun Xiong, Fuu-Jiun Hwang, Feng Sun, Yaowei Xie, Dacheng Mao, Geng-Lin Li, Guangyu Xu

**Affiliations:** 1 Department of Electrical and Computer Engineering, University of Massachusetts, Amherst, Massachusetts 01003 USA; 2 Department of Biology, University of Massachusetts, Amherst, Massachusetts 01003 USA

**Keywords:** Electrical and electronic engineering, Biomedical engineering

## Abstract

On-chip fluorescence imaging devices are recognized for their miniaturized and implantable nature that can benefit the study of intracellular dynamics at a variety of settings. However, it is challenging to integrate a spectral filter onto such devices (to block the excitation light) that has similar performance to the state-of-the-art emission filters used in fluorescence microscopes. In this work, we report a 100%-yield, spectrally filtered passive Si photodiode array designed for on-chip fluorescence imaging of intracellular Ca^2+^ dynamics. Coated with a spectral filter layer that has a high extinction ratio (>10^3^), our array features high wavelength selectivity (>10^2^), high linearity (*R*^2^ > 0.98), and low detection limit (45.1 μW 640/30 nm light). Employing fluorescence microscopy as the reference, we demonstrate that our array can conduct on-chip Ca^2+^ imaging in C2C12 cells that were chemically triggered to increase their intracellular Ca^2+^ levels. Importantly, our array-level data qualitatively captured the static fluorescence image of the cells and the intracellular Ca^2+^ dynamics, *both* of which are correlated with the microscope-collected data. Our results suggest the possible use of the spectrally filtered array towards a miniaturized on-chip fluorescence imaging device, which may open up new opportunities in tissue-level pharmaceutical screening and fundamental studies on cell networks.

## Introduction

Intracellular calcium concentration ([Ca^2+^]) plays important roles in the regulation of a variety of cellular functions^1,2^, such as muscle contraction^3,4^, neurotransmitter release^5,6^, and gene expression^7,8^. To monitor intracellular calcium dynamics in real time (i.e. Ca^2+^ imaging), fluorescence imaging has been broadly used for its outstanding sensitivity and specificity^9–11^. This method delivers fluorescent molecules (i.e. Ca^2+^ indicators) into living cells by either dye loading or gene expression^12,13^. These molecules specifically respond to the binding of Ca^2+^ ions, followed by a change in their emitted fluorescence intensity under an external light excitation.

Ca^2+^ imaging experiment is commonly conducted by fluorescence microscopy, which employs an objective lens and a digital camera to collect the fluorescence intensity emitted from living cells^14–16^. With the recent advent of integrated optoelectronics and optical materials, on-chip fluorescence imaging devices have emerged as viable alternatives for monitoring Ca^2+^ dynamics^17–20^. These devices are recognized for their miniaturized and implantable nature, which can ultimately offer deep tissue access for a variety of *ex vivo* or *in vivo* applications^21^. If successful, for instance, one may implant such devices to monitor the change of intracellular [Ca^2+^] during muscle recovery from injury^22^, or to conduct Ca^2+^ imaging of the neurocircuitry in deep brain^23^.

One key element in on-chip Ca^2+^ imaging devices is an integrated spectral filter, which serves to effectively block the excitation light from reaching to the photodetectors. As the excitation light intensity applied to Ca^2+^ indicators is a few orders higher than the emission light intensity from these indicators, the spectral filter must feature *both* high attenuation to the excitation light *and* high transmission to the emission light (i.e. high extinction ratio). To date, on-chip spectral filters have been made by Ag-SiO_2_ layers^24^, dielectric grating^25^, SiC alloy films^19,26^, or absorber-mixed polymer layers^20,27,28^. The latter two types of filters have been recently applied for on-chip fluorescence imaging of living cells^19,20^; however their extinction ratios are less than 10^2^, inferior to the state-of-the-art emission filters used in fluorescence microscopes.

Here we present 100%-yield, spectrally filtered passive Si photodiode (PD) array specifically designed for on-chip Ca^2+^ imaging. This array is integrated with a double-layered spectral filter, which is optimized to have >10^3^ extinction ratio for red-colored Ca^2+^ indicators (i.e. the ratio of the transmittance at the peak wavelengths of their emission and excitation spectra). We chose to build this device in a passive Si PD array structure for its CMOS compatibility, fast response, and low-power operation that is crucial for long-term use at *ex vivo* or *in vivo* settings^21^. Experimental results show that our PD array features high wavelength selectivity (>10^2^), high linearity (*R*^2^ > 0.98), and low detection limit (45.1 μW 640/30 nm light). We then demonstrate that our array can be applied for on-chip Ca^2+^ imaging of C2C12 cells, whose intracellular Ca^2+^ dynamics is commonly employed to study muscle contraction and regeneration^29–32^. The array-collected data qualitatively captured the static fluorescence image of the cells and the intracellular Ca^2+^ dynamics, *both* of which are correlated with the microscope-collected data. Our results suggest the possible use of the spectrally filtered passive Si PD array towards an on-chip fluorescence imaging device, which are generally applicable to monitor a variety of intracellular signals ultimately at *ex vivo* or *in vivo* settings.

## Results and Discussion

**Figure 1:**
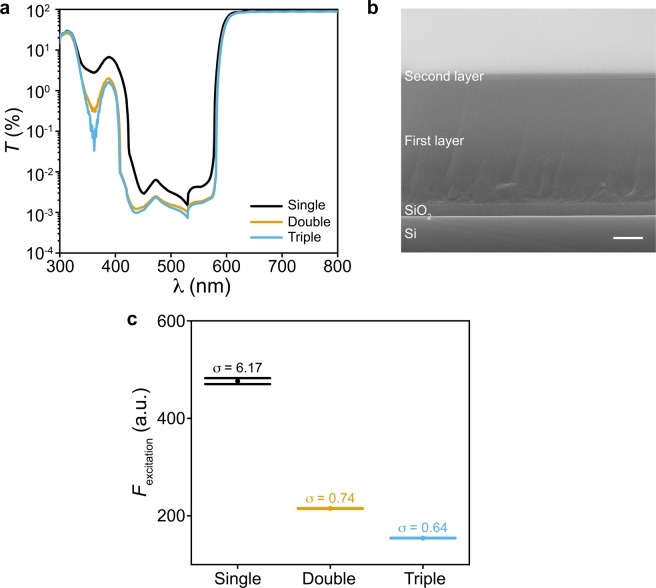
Optical properties of the spectral filter. (**a**) Transmission spectra of the single-, double- and triple-layered spectral filters. (**b**) The cross-sectional SEM image of a double-layered filter spin coated onto a Si/SiO_2_ substrate. Scale bar, 1 μm. (**c**) The normalized intensity of the excitation light leaked through the filter (*F*_excitation_) and its standard deviation (σ) measured from 500 frames of microscope data. The error bars represent ±1 s.d.

Our spectrally filtered Si PD arrays are based on 8-by-8 amorphous Si (α-Si) based p-i-n diodes routed in a cross-bar structure as we reported before^23,33^. Briefly, Si PDs are built from the plasma-enhanced chemical vapor deposition (PECVD) based α-Si layers, and contacted by indium tin oxide (ITO) and Cr/Au layers at their p- and n-terminals, respectively. Next, we chose to build the spectral filter by mixing visible light absorbing dyes into optically transparent photoresist (see Methods). This approach allows us to pattern the filter on top of the targeted PD pixels by photolithography, and to finetune its optical properties by adjusting the weight percentage (*wt*%) of the dyes and the photoresist thickness (*t*_photoresist_). Here we specifically design the filter to allow Ca^2+^ imaging with a cell-permeable Ca^2+^ indicator, X-Rod-1/AM, whose excitation and emission spectra peak at 580 nm and 601 nm, respectively^33,34,35^ (Supplementary Fig. S1). We found that an increase of the *wt*% of the dyes or *t*_photoresist_ will improve the extinction ratio, but at the expense of lower transmission to the emission light. As a result, two stacked photoresist layers (i.e. double-layer) mixed with 3.5 *wt*% of dyes result in the most balanced filter performance, >55% transmission at the peak of the emission spectrum and >10^3^ extinction ratio (Figs 1a, S1). These two photoresist layers were spin coated onto the PD array sequentially, with the second layer being much thinner than the first as shown in a scanning electron microscope (SEM) image (Fig. 1b). In addition, we found that adding a third photoresist layer on top does not significantly increase the extinction ratio (Fig. 1a) or reduce the standard deviation of the excitation light leaked through the filter measured from 500 frames of microscope data (Fig. 1c, see Methods); the latter would contribute to the background noise during Ca^2+^ imaging. For these reasons, we employed a double-layered filter on the PD array in this work.

**Figure 2:**
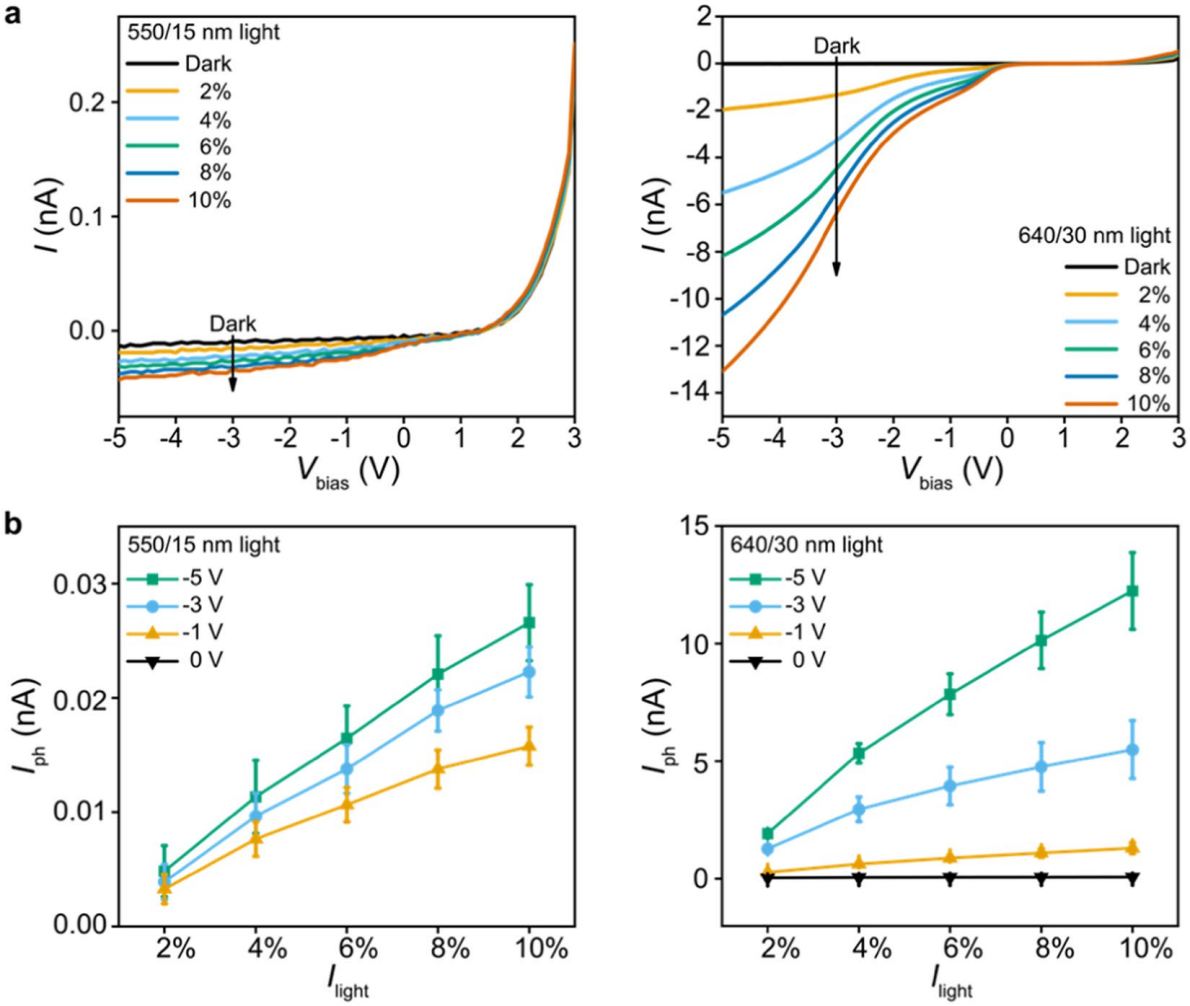
Wavelength selectivity and pixel-to-pixel variation. (**a**) Measured *I* − *V*_bias_ curves of a PD pixel at 550/15 nm (left) with *I*_light_ ranging from 5.2 mW (2%) to 26 mW (10%) and at 640/30 nm (right) with *I*_light_ ranging from 4.62 mW (2%) to 23.1 mW (10%), respectively. (**b**) *I*_ph_ vs. *I*_light_ with the pixel biased at *V*_bias_ = 0 V, −1 V, −3 V and −5 V and illuminated at 550/15 nm (left) and 640/30 nm (right) wavelengths. Statistics are based on measurements from eight pixels, four in the center and the other four at the corner of the 8-by-8 array. The error bars represent ±1 s.d.

We first examined the wavelength selectivity and the pixel-to-pixel variation of the fabricated array. To achieve this, we collected the *I*–*V* curves of eight filter-integrated pixels (Fig. 2a), four in the center and the other four at the corner of the array. Pixels are biased with *V*_bias_ ranging from −5 V to +3 V and illuminated at 550/15 nm and 640/30 nm wavelengths (the numbers before and after the slash are the center wavelength and the bandwidth of the light source, respectively). These two wavelengths were chosen to characterize the wavelength selectivity of the array as they fall into the excitation and emission spectra of the Ca^2+^ indicators, respectively (Supplementary Fig. S1). All eight pixels showed: (1) a rectified *I*–*V* as we expected in p-i-n structured PDs; and (2) a forward turn-on voltage around 1–1.5 V, which is likely due to the high doping levels in α-Si layers or the series resistance of the ITO contact lines^21^. To quantify the effect of the light power (*I*_light_), the wavelength, and the pixel bias (*V*_bias_) on the pixel response, we define the photocurrent (*I*_ph_) as the light-on current subtracted by the dark current (Fig. 2b). Consequently, the results show that *I*_ph_ at both wavelengths increases with *I*_light_, with the *I*_ph_ values at 640/30 nm being typically >10^2^ times larger than those at 550/15 nm with *V*_bias_ ranging from −1 V to −5 V. We note that this ratio of *I*_ph_ values at two wavelengths is less than the extinction ratio of the spectral filter (>10^3^), which may result from the inaccuracies in the low *I*_ph_ values measured at 550/15 nm (<30 pA). Moreover, we observe an increase of *I*_ph_ as the PDs are more negatively biased, which is likely due to the widening of the depletion width^36^. We thus chose *V*_bias_ = −5 V in the rest of this work for reasonably high *I*_ph_ values; a higher negative bias would further increase *I*_ph_, however at the expense of higher power consumption. Importantly, we find that at *V*_bias_ = −5 V the pixel-to-pixel variation among eight pixels (indicated by the error bars in Fig. 2b) is less than 8% when *I*_light_ at 640/30 nm is less than 10 mW (i.e. 2% and 4%). This result suggests good uniformity across the array in detecting the weak emission light during on-chip Ca^2+^ imaging.

**Figure 3:**
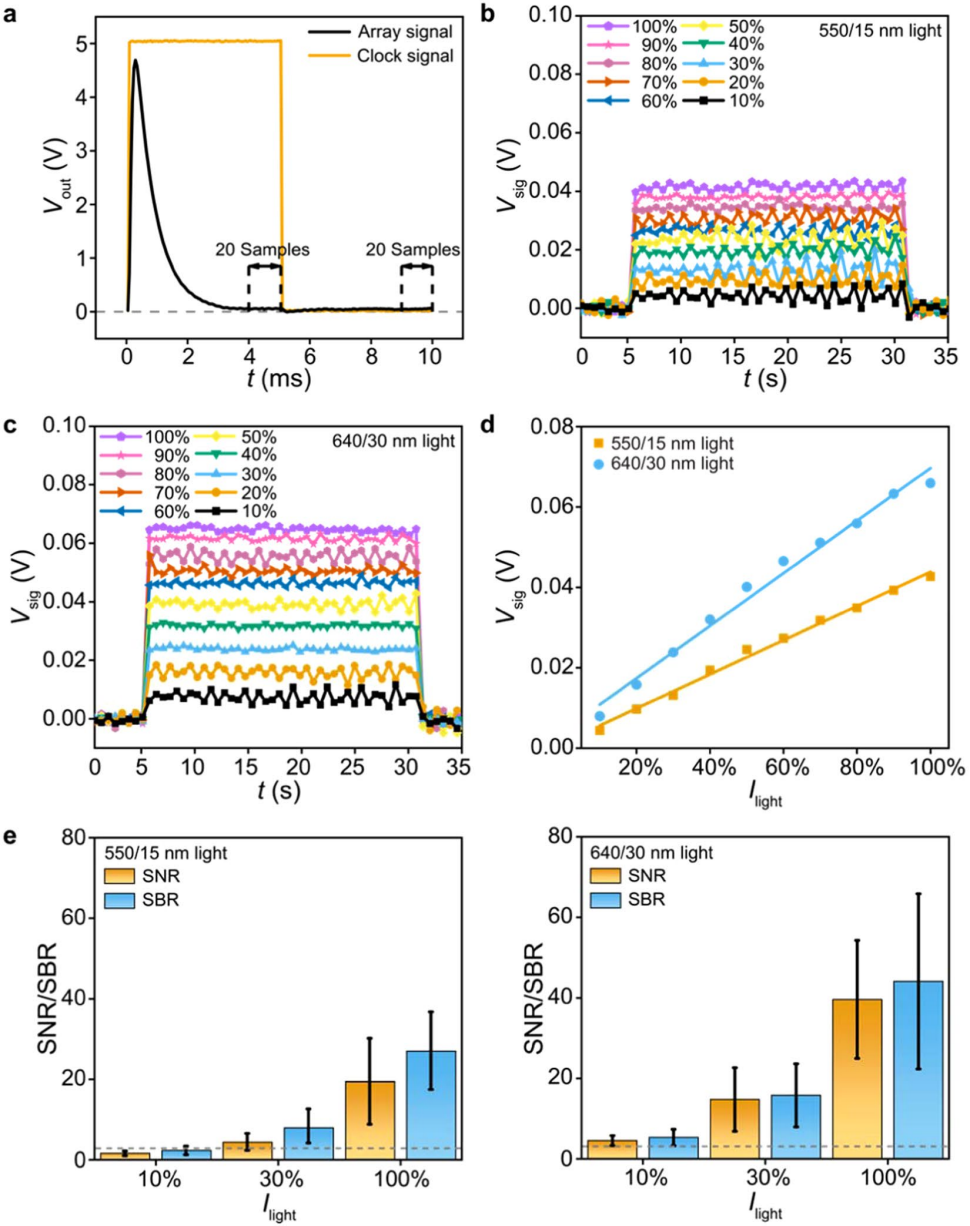
Linearity and limit of detection. (**a**) Measured 10 ms *V*_out_-trace of one pixel in the dark (50 μs per sample) and the clock signal employed for array scanning. The pixel signal (*V*_sig_) during each frame of the array is averaged from two 1-ms steady-state data in the *V*_out_-trace (i.e. a total of 40 samples marked by dashed lines). (**b**) Measured 40 frames of *V*_sig_ values when the array is illuminated at 550/15 nm with *I*_light_ ranging from 26 mW (10%) to 260 mW (100%). (**c**) Measured 40 frames of *V*_sig_ values when the array is illuminated at 640/30 nm with *I*_light_ ranging from 45.1 μW (10%) to 451 μW (100%). (**d**) *V*_sig_ (40-frame average) vs. *I*_light_ under 550/15 nm and 640/30 nm illuminations. (**e**) Measured SNR and SBR values under 550/15 nm (left, 260 mW at 100%) and 640/30 nm (right, 451 μW at 100%) illuminations. Dashed lines indicate SNR = 3 and SBR = 3. Statistics are based on measurements from eight pixels, four in the center and the other four at the corner of the 8-by-8 array. The error bars represent ±1 s.d.

To characterize the device at array level, we built an off-board multiplexing circuit to sequentially bias the select PD pixels at *V*_bias_ = −5 V during each frame of the array, with the unselected pixels left open-circuited (Supplementary Fig. S2). Specifically, we use two analog multiplexers for row- and column-scanning, two counters for decoding the clock signal (employed for array scanning), and one transimpedance preamplifier to amplify the pixel current into a voltage readout (*V*_out_), followed by a 50/60 Hz noise eliminator to remove the noise from the power line (see Methods). With this noise removal, we sequentially record a 10 ms *V*_out_-trace from each pixel using a digital oscilloscope that has 256 MS buffer memory (50 μs per sample, see Methods). We chose this 10 ms sampling time per pixel because the collected *V*_out_-traces typically take >3 ms to settle following the rising and falling edges of the clock signal (Fig. 3a, measured in the dark). This settling may result from the fast change in the output signals of multiplexers or counters, which would introduce transient charging/discharging currents through the capacitance of the select PD. Importantly, we adjusted the compensation capacitance across the preamplifier to shorten this settling time, and averaged two 1-ms steady-state data in the recorded *V*_out_-trace (i.e. one 1-ms data in each half of the trace, 40 samples in total) as the pixel signal (*V*_sig_) during each frame of the array.

We next quantify the *V*_sig_ values at various *I*_light_ values to examine the light-detection limit of the array. To achieve this, we collected 40 frames of data (each frame takes 640 ms to scan over the array, see Supplementary Fig. S3) with the array being illuminated at: 1) 550/15 nm with *I*_light_ ranging from 26 mW (10%) to 260 mW (100%), selected to quantify the background noise contributed by the excitation light leaked through the filter; and 2) 640/30 nm with *I*_light_ ranging from 45.1 μW (10%) to 451 μW (100%), selected to evaluate if the array can detect the weak emission light. Here the weak 640/30 nm light power is achieved by applying 512-time attenuation in the light path, which is offered by neural-density filters on the microscope (i.e. *I*_light_ ranges from 23.1 mW to 231 mW before attenuation). The data show that *V*_sig_ values under attenuated 640/30 nm illumination are still larger than those under non-attenuated 550/15 nm illumination (Fig. 3b,c). This result suggests that our array features >10^2^ wavelength selectivity, which originates from the high extinction ratio of the filter.

From these 40 frames of *V*_sig_ data, we found good linearity in *V*_sig_ − *I*_light_ relationships (*R*^2^ > 0.98) at *both* 550/15 nm *and* 640/30 nm wavelengths (Fig. 3d). Furthermore, we made statistical analysis of the signal-to-noise ratio (SNR) and the signal-to-background ratio (SBR) from the eight pixels described in Fig. 2; here SNR [SBR] is defined as the mean *V*_sig_ value when light is on divided by its standard deviation [divided by the standard deviation of the *V*_sig_ value in the dark]. The data show that the 640/30 nm illumination results in higher SNR and SBR than the 550/15 nm illumination (Fig. 3e), suggesting that our array can detect the emission light with statistically significant *V*_sig_ data. Importantly, the SNR and SBR values at 640/30 nm illumination are larger than 3 for *I*_light_ down to 45.1 μW (10%), suggesting the low light-detection limit of the array.

**Figure 4:**
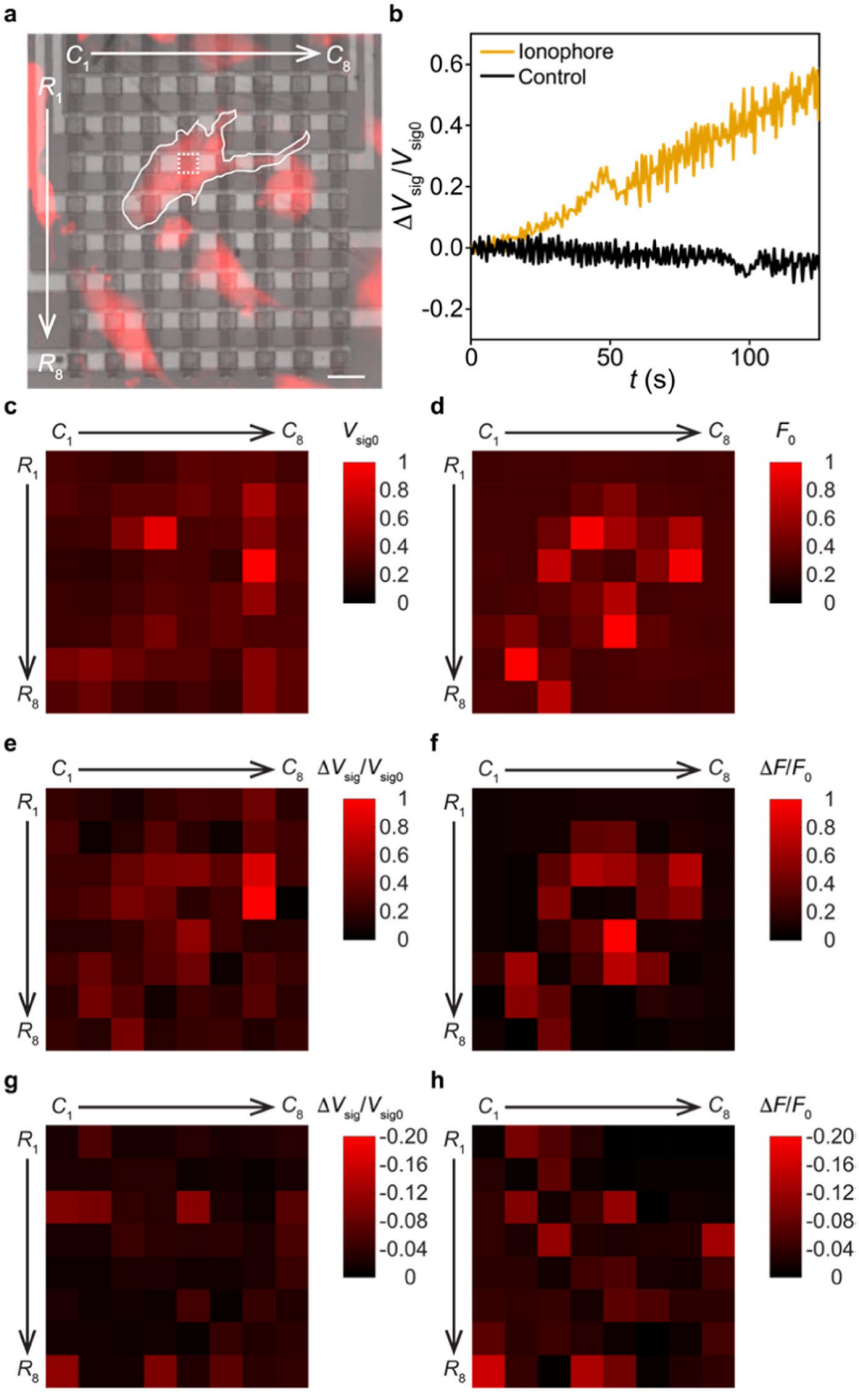
On-chip Ca^2+^ imaging in C2C12 cells. (**a**) Fluorescence image of C2C12 cells overlaid with the image of the filter-integrated array (false coloured, *R*_1_ − *R*_8_ and *C*_1_ − *C*_8_ are the row and column numbers of the array). One PD pixel with one cell right on top (outlined) is squared for data analysis in b. Scale bar, 20 μm. (**b**) Measured Δ*V*_sig_/*V*_sig0_ traces when 100 μM A23187 ionophores (yellow) or only DMSO-premix (black) were added to the cells. The two Δ*V*_sig_/*V*_sig0_ traces are from the PD pixels (right below the targeted cells) squared in a and Supplementary Fig. S5g, respectively. (**c**) Array-collected *V*_sig0_-mapping data after calibrating out the pixel-to-pixel variation. (**d**) Microscope-collected *F*_0_-mapping data. The presented *F*_0_ values are averaged from the region of each PD pixel in a. (**e**) Array-collected Δ*V*_sig_/*V*_sig0_-mapping data from the ionophore adding experiment (after calibrating out the pixel-to-pixel variation). (**f**) Microscope-collected Δ*F/F*_0_-mapping data from the ionophore adding experiment. The presented Δ*F/F*_0_ values are averaged from the region of each PD pixel in a. (**g**) Array-collected Δ*V*_sig_/*V*_sig0_-mapping data from the control experiment (after calibrating out the pixel-to-pixel variation). The absolute values of the presented data are in the same scale of e. (**h**) Microscope-collected Δ*F/F*_0_-mapping data from the control experiment. The absolute values of the presented data are in the same scale of (**f**).

To demonstrate the on-chip Ca^2+^ imaging in C2C12 cells, we placed a 100-μm thick glass slide on top of the PD array to prevent the extracellular solution from shortening pixels (Supplementary Fig. S4). An upright fluorescence microscope was then employed to conduct fluorescence microscopy, which serves as a reference to the on-chip imaging data collected by our array; the 550/15 nm excitation light power is set as 26 mW (10%) to alleviate the photobleaching effect of the Ca^2+^ indicators^37^. On the biology side, we seeded C2C12 cells on coverslips, cultured them at 37 °C in a humidified incubator, and added X-Rod-1/AM to the cell culture before the imaging experiment, all following the manufacturers’ recommended protocols (see Methods). After these preparation steps, we placed the coverslip (with cells facing up) on the glass slide, and aligned cells to the array position (Fig. 4a). Subsequently, we applied extra extracellular solution on top of the cells, which serves to work with a water-immersion objective lens and prevent cells from getting dry during the experiment.

During the Ca^2+^ imaging experiment, we added A23187 ionophores – premixed with dimethyl sulfoxide (DMSO) and pluronic F127 solvent (see Methods) – to the extracellular solution to reach a final concentration of 100 μM, which work to bring extracellular Ca^2+^ ions into the cell and increase the intracellular Ca^2+^ level^38,39^. We found that these ionophores do induce significant increase in the recorded *V*_sig_ values (subtracted by the background value measured in the dark, i.e. no cells on top of the array) from pixels beneath the cell (Fig. 4b). Normalized by the *V*_sig_ value measured right after adding ionophores (i.e. *V*_sig0_ at *t* = 0), the resulting Δ*V*_sig_/*V*_sig0_ trace is in good contrast with that collected in the control experiment, when only DMSO and pluronic F127 solvents were added. This result suggests that the *V*_sig_ increase detected by our pixels does specifically reflect the intracellular Ca^2+^ dynamics caused by ionophores.

To validate our results at the array level, we generate a normalized *V*_sig0_ -mapping plot across the entire array, serving to visualize the cell positions on top of the PD pixels. To remove the effect of the pixel-to-pixel variation on the results, at each pixel we divide its background-subtracted *V*_sig0_ value (Fig. 4b) by its mean response to 45.1 μW 640 nm illumination (from 40 frames of data in Fig. 3c); the resulting *V*_sig0_ -mapping data thus exclude the fixed pattern noise among pixels (Fig. 4c), and can be viewed as the mapping of the cell-emitted fluorescence intensities (*F*) captured by our array. For comparison purposes, we also generate a normalized *F*_0_-mapping plot from the microscope data measured at *t* = 0, where the presented *F*_0_ values (subtracted by the background value measured at the dark region in the field-of-view) are averaged from the region of each PD pixel in the microscope image (Fig. 4d). The results show that the *V*_sig0_-mapping data are qualitatively correlated with the *F*_0_-mapping data with the Pearson correlation coefficient *r* ~ 0.46, suggesting that our array can capture the *static* fluorescence image of cells. We repeated this comparison in a total of three experiments, two with the ionopore adding and one with the control, all of which show such qualitative correlation between the *V*_sig0_- and *F*_0_-mapping data (Supplementary Fig. S5).

Taking one step further, we now examine the intracellular Ca^2+^ dynamics captured by our array. To achieve this, we chose to map the Δ*V*_sig_/*V*_sig0_ values measured at *t* ~ 125 s (Fig. 4b) to visualize the Ca^2+^ signaling in cells on the array. To calibrate out the fixed pattern noise, at each pixel we divide its Δ*V*_sig_/*V*_sig0_ value at *t* ~ 130 s by its *normalized differential response* to the low-level 640/30 nm illumination, defined as (*V*_sig @ 20%_ − *V*_sig @ 10%_)/*V*_sig @ 10%_ obtained from Fig. 3c (averaged from 40 frames of data); the resulting Δ*V*_sig_/*V*_sig0_ mapping data can thus be viewed as the mapping of the increase of intracellular Ca^2+^ levels captured by our array (Fig. 4e). For comparison purposes, we also generate a normalized Δ*F/F*_0_-mapping plot from the microscope data measured at *t* ~ 130 s, where the presented Δ*F/F*_0_ values (using *F*_0_ values obtained in Fig. 4c) are averaged from the region of each PD pixel in the microscope image (Fig. 4f). The results show that the Δ*V*_sig_/*V*_sig0_-mapping data are qualitatively correlated with the Δ*F/F*_0_-mapping data with *r* ~ 0.68, suggesting that our array can conduct on-chip fluorescence imaging of the intracellular Ca^2+^ dynamics. We repeat this comparison in two independent experiments, both of which show such qualitative correlation between the Δ*V*_sig_/*V*_sig0_- and Δ*F/F*_0_-mapping data (Supplementary Fig. S5). In contrast, the Δ*V*_sig_/*V*_sig0_- and Δ*F/F*_0_-mapping plots from the control experiment are mostly low signals (Fig. 4g,h with *r* ~ 0.60, the absolute values of the presented data are in the same scale of Fig. 4e,f), since no ionophores were added to increase the intracellular Ca^2+^ levels.

We noted that the deviation of the array data from the microscope data (i.e. *V*_sig0_ vs. *F*_0_ and Δ*V*_sig_/*V*_sig0_ vs. Δ*F/F*_0_) may result from the fact that the microscope and the array collect cell-emitted lights via different optical paths. Specifically, the microscope collects the cell-emitted light that enters into the objective lens, whereas the array collects the cell-emitted light that passes through *both* the coverslip *and* the glass slide, which may cause scattering of the light (see Supplementary Fig. S4). Also, since cells emit light omnidirectionally, the spatial contrast in *V*_sig0_ and Δ*V*_sig_/*V*_sig0_ data, collected by our array in a lens-free manner, is not as good as the microscope data. This issue is intrinsic to the on-chip imaging setting^17,18^, and can be alleviated by designing angular selective pixels in the future^40^.

Finally, we remark that for *ex vivo* or *in vivo* applications our spectrally filtered PD arrays will need to be passivated with biocompatible films (e.g. spin-coated SU-8 layers) to prevent cells from direct contacting the photoresist based spectral filter. The passivated array is expected to feature enhanced device biocompatibility and help avoid filter degradation in the biological environment. For instance, such passivated PD array can be built along a solid-state shank with a comparable dimension to that of the implantable silicon microelectrode arrays^41^; the resulting device may ultimately enable *in vivo* optical neural recording in head-fixed animals with the excitation light being provided by two-photon microscopy^16,21^.

## Conclusions

In sum, we presented a 100%-yield, spectrally filtered passive Si PD array designed for on-chip fluorescence imaging of intracellular Ca^2+^ dynamics. Based on optoelectrical characterizations at both pixel and array levels, the fabricated array features high wavelength selectivity (>10^2^), high linearity (*R*^2^ > 0.98), and low detection limit (45.1 μW 640/30 nm light). Employing fluorescence microscopy as the reference, we demonstrate that our array can be employed to conduct on-chip Ca^2+^ imaging in C2C12 cells that were chemically triggered to increase their intracellular Ca^2+^ levels. The data show that the array-collected data qualitatively captured the *static* fluorescence image of cells and the intracellular Ca^2+^ dynamics, both of which were evidenced by the microscope-collected data. Our results suggest the possible use of the spectrally filtered array towards a miniaturized on-chip fluorescence imaging device. Combined with its fast response, good scalability, and low power consumption, such device platform may open up new opportunities in pharmaceutical screening at the tissue level as well as fundamental studies on cell networks ultimately at *ex vivo* or *in vivo* settings.

## Methods

### Spectrally filtered Si PD array fabrication

We first pattern Cr contacts on a Si/SiO_2_ substrate to contact with n-doped α-Si. Then, n-doped (n, 40 nm), un-doped (i, 600 nm), and p-doped (p, 40 nm) α-Si layers are sequentially deposited by 250 °C PECVD steps. After PECVD steps, PD arrays are patterned into 12 μm-sized pixels (with a 20 μm pitch) by reactive-ion etching, and passivated with 150 nm SiO_2_ film by PECVD at 300 °C. We then applied buffered oxide etching to create contact openings, followed by sputtering ~120 nm ITO to contact with p-doped α-Si. Finally, Cr/Au layers (10/200 nm) is deposited on the pad area for wire-bonding. The spectral filter was made by mixing absorbing dyes, Epolight 5391 and Epolight 5843 (Epolin), into photoresist NR9-1000PY (Futurrex), sequentially spin-coated (1000 rpm, 40 s) twice onto the PD array to form double layers, and patterned by photolithography all at once. The resulting array is wire-bonded onto a printed circuit board.

### Adding Ca^2+^ indicators to the C2C12 cell culture

C2C12 cells (CRL-1772, ATCC) were cultured in Dulbecco’s modified Eagle medium (Gibco) with 10% fetal bovine serum (Corning) and 100 units/mL penicillin-streptomycin (Gibco) at 37 °C and 5% CO_2_ in a humidified incubator. Glass coverslips were cut into small pieces, sterilized, air dried, and placed into different wells of a 48-well plate. Cells were then seeded onto these coverslips, one in each well, at a density of 0.25 × 10^5^/cm^2^ and used within 2 days. Before conducting the imaging experiment, cells were first three-time washed by an extracellular solution that consists of 140 mM NaCl, 5.4 mM KCl, 1.8 mM CaCl_2_, 1.8 mM MgCl_2_, 11 mM glucose, and 10 mM HEPES (pH ~ 7.4). Then, cell-permeable X-Rhod-1/AM (Thermal Fisher Scientific), premixed in DMSO and pluronic F-127 (Sigma) in a 1:1: volume ratio, were added to the extracellular solution at a final concentration of 7.5 μM for 45 minutes at 37 °C in the dark. Cells were then three-time washed again by the extracellular solution to remove the extracellular X-Rhod-1/AM.

### Ca^2+^ imaging experiment

Cells loaded with X-Rhod-1/AM (together with the coverslip) are placed on top of the array (covered by a 100 μm glass slide), with ~45 μL extracellular solution added to work with the water immersion objective. On the ionophore side, A23187 stocks (Invitrogen, 20 mM in DMSO) were premixed with equal volume of pluronic F-127, and diluted to 1 mM by the extracellular solution. During the imaging experiment, 5 μL of this A23187 premix was added to the 45 μL extracellular solution (at a final concentration of 100 μM), serving to increase the intracellular Ca^2+^ levels. In the control experiment, DMSO were premixed with equal volume of pluronic F-127; 5 μL of such DMSO-premix was added to the 45 μL extracellular solution during the experiment.

Fluorescence imaging of C2C12 cells was performed using an epifluorescence upright microscope (FN1, Nikon) equipped with a Zyla4.2 plus sCMOS (scientific complementary metal-oxide semiconductor) camera (Andor, USB 3.0) and a SPECTRA X light engine (Lumencor). NIS-Elements Advanced Research software (Nikon) was used for automated microscope and camera control. Cells were imaged with a CFI6O Fluor 20× water immersion objective lens (NA = 0.5, Nikon) at room temperature (100 ms exposure time per frame, no binning). We applied a 550/15 nm excitation light (with 26 mW at 10%), a 585 nm long-pass dichroic mirror, and a 632/60 nm emission filter during the Ca^2+^ imaging experiment.

### Device characterization

To characterize the optical properties of the spectral filters (Fig. 1), we spin coated them onto a 100 μm-thick coverslip (Fisher Scientific) and measure their transmission spectra by an UV-vis-NIR spectrometer (SHIMADZU 3600). The intensity fluctuation of the 550/15 nm excitation light leaked through the filters was measured by an epifluorescence upright microscope (BX51WIF, Olympus) equipped with a 138 Retiga 2000R CCD (charge-coupled device) camera (QImaging) and a Lambda DG-4 light source (Sutter Instrument). The *F*_excitation_ values were measured from 500 frames of data (100 ms exposure time per frame, no binning) with a UPLFLN 4× objective lens (NA = 0.13, OLYMPUS). The SEM image was taken by a thermal field emission scanning electron microscope (JSM-7001F, JEOL).

The *I*–*V* curves of PD pixels were measured by Keysight B1500A with a 100 mV sweep step of *V*_bias_; pixels were illuminated at 550/15 nm (260 mW at 100%) or 640/30 nm (231 mW at 100%) wavelengths, provided by FN1 through a CFI6O Plan Achromat 10× objective lens (NA = 0.25, Nikon) (Fig. 2). At the array level (Figs 3 and 4), we built an off-board multiplexing circuit in a home-made faradic cage, using two ADG406 multiplexers and two CD4029BMS counters to select pixels and bias them at *V*_bias_ = −5 V with Keysight B2902A. The pixel current is amplified to a voltage readout, *V*_out_, by a low-noise preamplifier SR570 (Stanford Research Systems), followed by a Hum bug noise eliminator (A-M Systems) to remove the 50/60 Hz noise. The preamplifier operated in the low noise mode, with a 10 nA/V sensitivity, and a 3 kHz cutoff frequency of a low-pass signal filter (12 dB/octave to reduce the high-frequency noise in the recorded *V*_out_ -trace), all of which were optimized to maximize the SNR and SBR obtained from the 40 frames of *V*_sig_ data (Fig. 3). The resulting *V*_out_-trace and the clock signal are recorded by an 8-channel deep-memory digital oscilloscope (Pico 4824).

## Supplementary information

Supplementary Information

## Data Availability

All data generated or analyzed during this study are included in this published article and its Supplementary Information.
