# Author Correction: Spectrally filtered passive Si photodiode array for on-chip fluorescence imaging of intracellular calcium dynamics

**DOI:** 10.1038/s41598-020-60962-y

**Published:** 2020-02-26

**Authors:** Zheshun Xiong, Fuu-Jiun Hwang, Feng Sun, Yaowei Xie, Dacheng Mao, Geng-Lin Li, Guangyu Xu

**Affiliations:** 1 Department of Electrical and Computer Engineering, University of Massachusetts, Amherst, Massachusetts 01003 USA; 2 Department of Biology, University of Massachusetts, Amherst, Massachusetts 01003 USA

Correction to: *Scientific Reports* 10.1038/s41598-019-45563-8, published online 24 June 2019

This Article contains errors in the reference list where references 1-3 should be listed as references 9-11, and references 4-11 should be listed as references 1-8.

Additionally, there is an error in the Author Contributions where,

“Z.X. and F.-J.H. performed the Ca2^+^ imaging experiments.”

should read:

“Z.X. and F.-J.H. performed the Ca^2+^ imaging experiments.”

