# Supplementary material for: Spectrally filtered passive Si photodiode array for on-chip fluorescence imaging of intracellular calcium dynamics
Source: Sci Rep. 2019 Jun 24;9:9083. doi: 10.1038/s41598-019-45563-8 (PMC6591417; doi:10.1038/s41598-019-45563-8)

Supplementary Information

Spectrally filtered passive Si photodiode array for on-chip fluorescence imaging of intracellular calcium dynamics

Zheshun Xiong[1, +], Fuu-Jiun Hwang[2, +], Feng Sun[1], Yaowei Xie[1], Dacheng Mao[1], Geng-Lin Li[2, *] & Guangyu Xu[1, *]

[1]*Department of Electrical and Computer Engineering, University of Massachusetts, Amherst, Massachusetts 01003, USA.*

[2]*Department of Biology, University of Massachusetts, Amherst, Massachusetts 01003, USA.*

[+] These authors contributed equally to this work.

* Corresponding Authors; (e-mail): guangyux@umass.edu, genglin@bio.umass.edu.

Supplementary Figure S1 | Filter design, including the transmission spectrum of the double-layered filter (left), the excitation and emission filters employed in the microscope (left), and the excitation /emission spectra of X-Rod-1/AM (right).

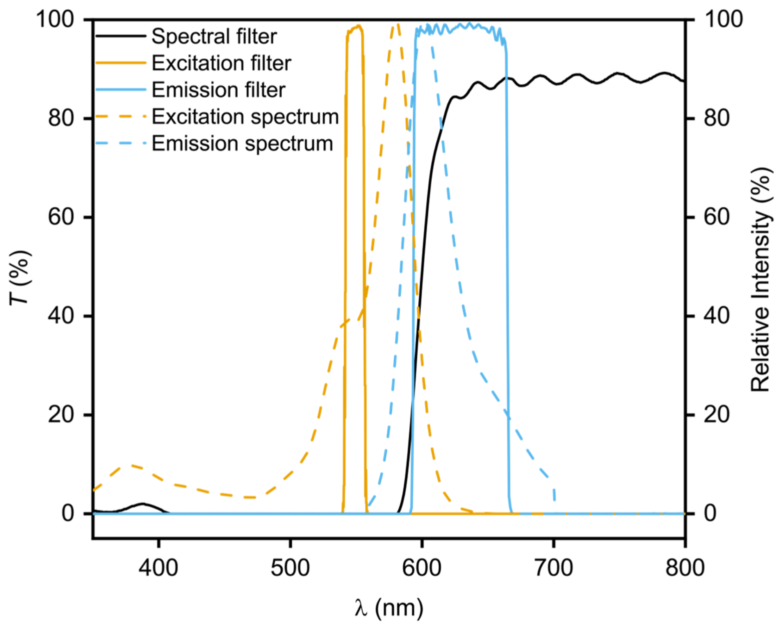

Supplementary Figure S2 | Off-board multiplexing circuits for scanning over the 8-by-8 PD array (SMU: source-measurement unit, Keysight B2902A).

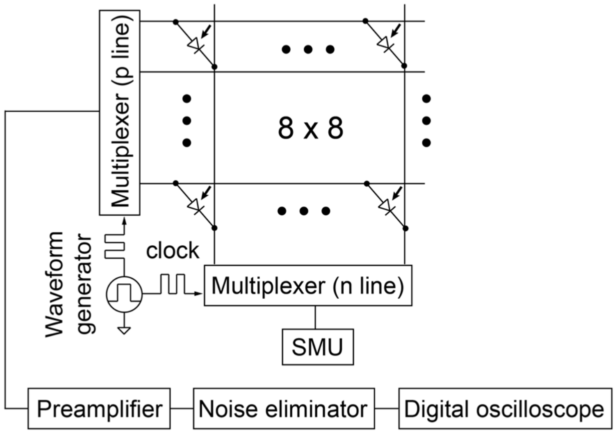

Supplementary Figure S3 | Single-frame data of all 64 pixels measured under 45. 1 μW (10%) and 90.2 μW (20%) 640/30 nm illumination and in the dark. These data were used to calibrate out the pixel-to-pixel variation.

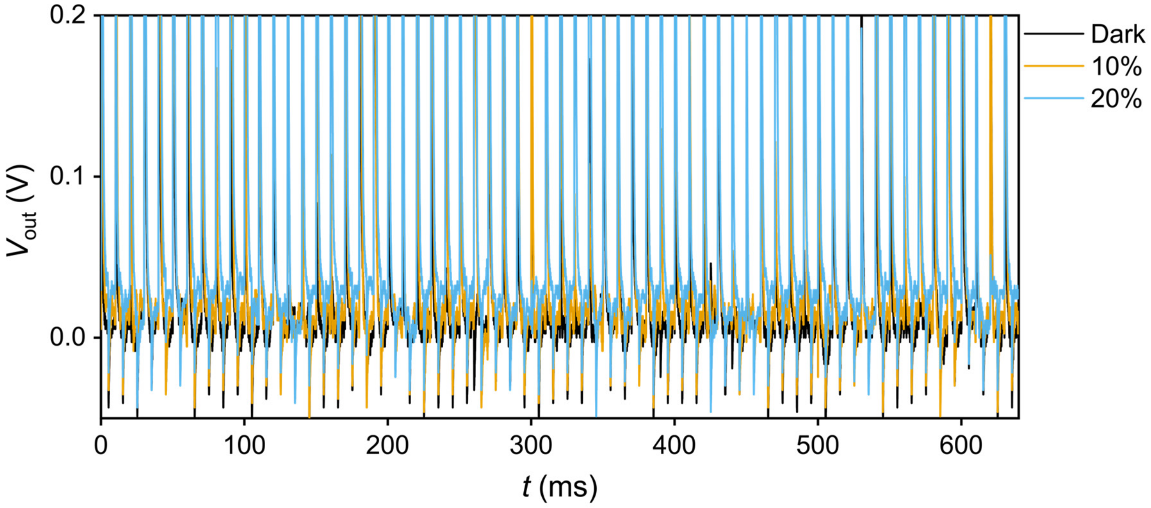

Supplementary Figure S4 | Testing setup for on-chip Ca^{2+} imaging.

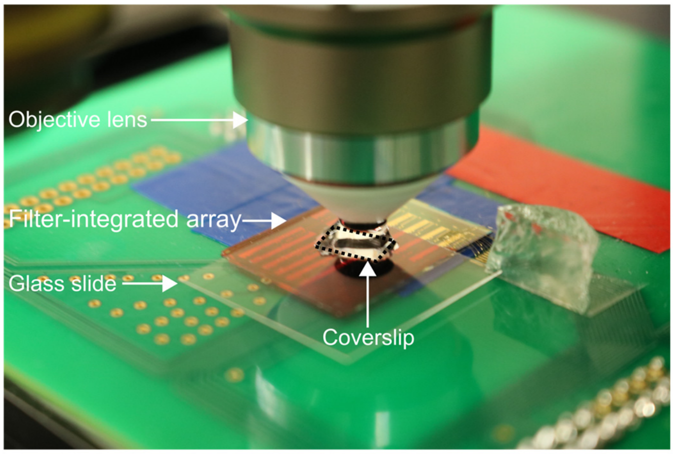

Supplementary Figure S5 | Additional experimental results from another ionopore adding experiment (a-f, left) and a control experiment with only DMSO-premix adding (g-i, right). The two $\Delta V_{sig}/V_{sig0}$ traces in Fig. S5b are from the PD pixels (right below the targeted cells) squared in Figs. S5a and S5g, respectively. For this additional ionopore adding experiment, the presented $\Delta V_{sig}/V_{sig0}$ and $\Delta F/F_0$ values are measured at $t \sim 140$ s; for the control experiment, the $\Delta V_{sig}/V_{sig0}$ and $\Delta F/F_0$ data are presented in Figs. 4g. and 4f in the main text. Scale bar, 20 µm. The r values are ~ 0.57 between Figs. S5c and S5d, ~ 0.49 between Figs. S5e and S5f, and ~ 0.38 between Figs. S5h and S5i, respectively.

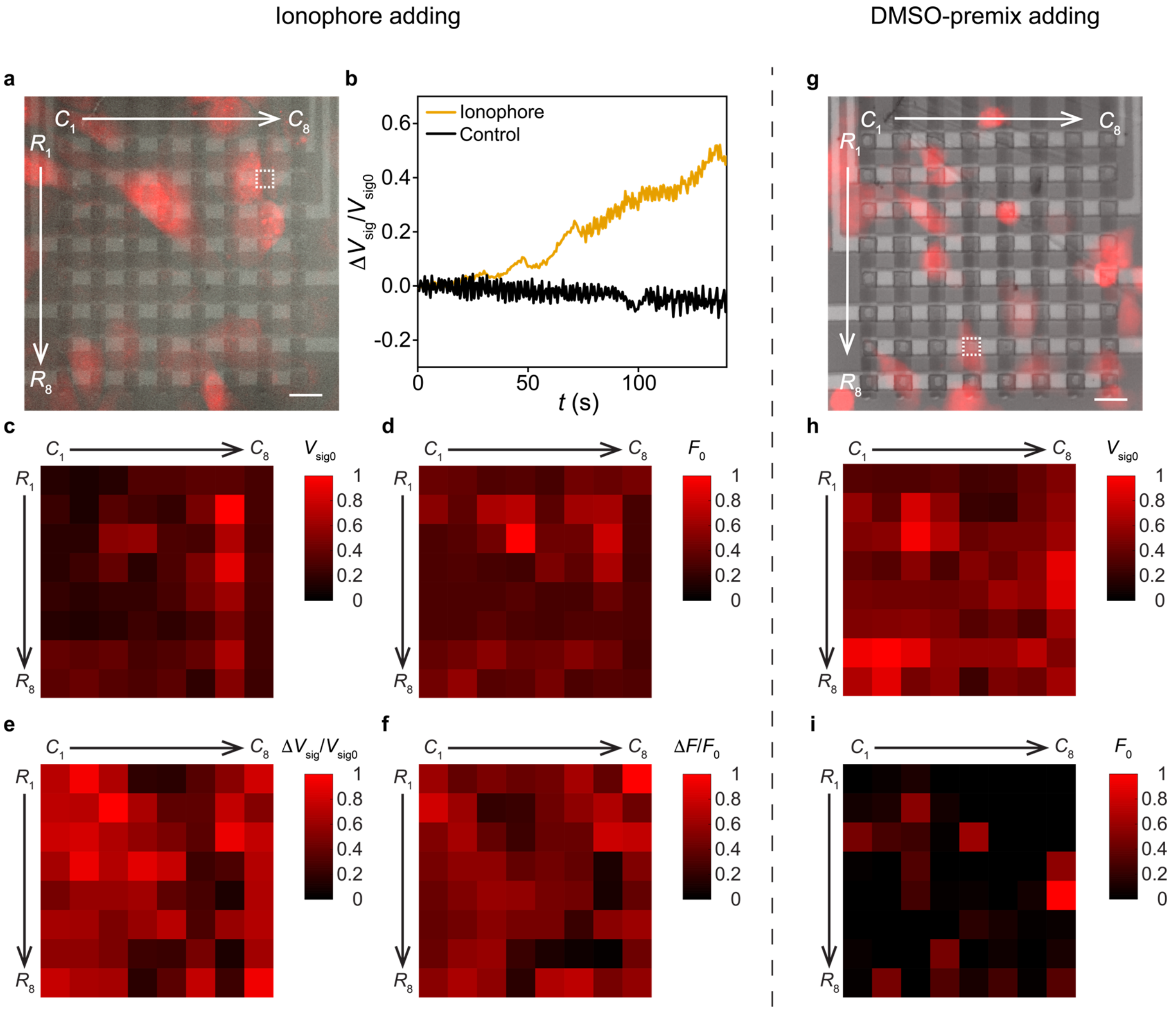

Supplement: Supplementary file 1 — Supplementary Information [file 41598_2019_45563_MOESM1_ESM.pdf]
